# Music-based therapy in rehabilitation of people with multiple sclerosis: a systematic review of clinical trials

**DOI:** 10.1590/0004-282X-ANP-2020-0374

**Published:** 2021-06-23

**Authors:** Josiane Lopes, Ivo Ilvan Keppers

**Affiliations:** 1 Universidade Estadual do Centro-Oeste Departamento de Fisioterapia Guarapuava PR Brazil Universidade Estadual do Centro-Oeste, Departamento de Fisioterapia, Guarapuava PR, Brazil.

**Keywords:** Multiple Sclerosis, Music Therapy, Rehabilitation, Esclerose Múltipla, Musicoterapia, Reabilitação

## Abstract

**Background:**

Multiple sclerosis (MS) is a major cause of chronic neurological disability in young adults. An increasing number of controlled studies have assessed the potential rehabilitative effects of new drug-free treatments, complementary to the standard care, including music-based therapy (MBT).

**Objective:**

To analyze the evidence for the effectiveness of MBT within the therapeutic approaches to individuals diagnosed with MS.

**Methods:**

A systematic review of clinical trials was performed with searches in the following databases: BIOSIS, CINAHL, Cochrane, EBSCO, ERIC, Google Scholar, IBECS, LILACS, LISA (ProQuest), Medline, PEDro, PsycINFO (APA), Psychological & Behavioral, PubMed, SciELO, Scopus, SPORTDiscus and Web of Science. Clinical trials comparing MBT *versus* conventional therapy/no intervention were included.

**Results:**

From the 282 studies identified, 10 trials were selected. Among these, the total sample consisted of 429 individuals: 253 were allocated to the experimental group (MBT) and 176 to the control group (conventional therapies or no intervention). All the studies presented high methodological quality. Modalities of MBT were clustered into four groups: (1) Rhythmic auditory; (2) Playing musical instruments; (3) Dance strategy; and (4) Neurological music therapy. Overall, the studies consistently showed that MBT was better than conventional therapy or no intervention, with regard to gait parameters (double support time and walking speed), fatigue level, fatigability, coordination, dexterity, balance, walking endurance, lower extremity functional strength, emotional status and pain. Regarding mental fatigability and memory, the data were conflicting and the evidence was unclear.

**Conclusion:**

MBT is a safe and effective approach for clinical rehabilitation of MS patients that leads to positive results regarding both motor and non-motor functions.

## RESUMO

**Introdução:**

A esclerose múltipla (EM) é a maior causa de incapacidade neurológica crônica em adultos jovens. Um crescente número de estudos controlados avalia a reabilitação por meio de tratamentos não medicamentosos, cuidados complementares incluindo a terapia baseada na música (TBM).

**Objetivo:**

Analisar a evidência de efetividade da TBM na abordagem terapêutica de indivíduos diagnosticados com EM.

**Métodos:**

Foi realizada uma revisão sistemática de ensaios clínicos com busca nas bases de dados Biosis, Cinahl, Cochrane, Ebsco, Eric, Google Scholar, Ibecs, Lilacs, Lisa (ProQuest), Medline, PEDro, PsycINFO (APA), Psychological & Behavioral, PubMed, SciELO, Scopus, Sportdiscus e Web of Science. Foram incluídos ensaios clínicos comparando TBM *versus* terapia convencional/sem intervenção.

**Resultados:**

Dentre os 282 estudos identificados, 10 ensaios foram selecionados. Desses, a amostra total consistia em 429 indivíduos (253 alocados no grupo experimental (TBM) e 176 no grupo controle (terapias convencionais ou sem intervenção). Todos os estudos apresentaram elevada qualidade metodológica. Modalidades da TBM foram reunidas em quatro grupos: (1) Audição rítmica; (2) Tocar instrumentos musicais; (3) Dança como estratégia; e (4) Terapia neurofuncional baseada na música. A maioria dos estudos identificou que a TBM é melhor que a terapia convencional ou nenhuma intervenção nos parâmetros da marcha (duplo apoio e velocidade), níveis de fadiga, fatigabilidade, coordenação, destreza, equilíbrio, força de membros inferiores, estado emocional e dor. Os dados sobre fatigabilidade mental e memória foram conflitantes e as evidências são incertas.

**Conclusão:**

A TBM é uma abordagem segura na reabilitação clínica de indivíduos com EM e propicia resultados positivos nas funções motoras e não motoras.

## INTRODUCTION

Multiple sclerosis (MS) is an autoimmune and neurodegenerative disorder characterized by destruction of myelin in the central nervous system (CNS), caused by a complex interplay between genetic and environmental factors^1^^,^^2^. MS is mostly diagnosed in young adults, with disease onset occurring in most cases between the ages of 20 and 40 years^3^^,^^4^^,^^5^. There is increasing incidence and prevalence of MS in both developed and developing countries. It affects approximately 2.3 million people worldwide (one million only in USA)^6^. However, the underlying cause of MS remains uncertain^7^.

It is a major cause of chronic neurological disability in young adults (aged 18–50 years), associated with complex disabilities^8^. MS can affect any area of the CNS with variable clinical manifestations (e.g. visual, motor and sensory deficits, speech disturbances, sphincter disorders, cognitive impairment, sexual problems and fatigue)^9^^,^^10^. These disabilities usually lead to progressive limitation of functioning in daily life, thus requiring longer-term multidisciplinary management. MS has a variable and uncertain prognosis^11^^,^^12^. To date, the aim of the pharmacological treatments available for MS, and in particular the disease-modifying treatments, is to achieve reduction of the clinical relapse severity and frequency, thereby slowing down the disease progression^13^.

Nevertheless, many of these symptoms can deleteriously impact the occupational profile, social participation, self-esteem and quality of life of patients. Over the last 11 years, an increasing number of controlled studies conducted on several neurological disorders have assessed the potential rehabilitative effects of new drug-free treatments that would be complementary to the standard care, including music-based therapy (MBT)^3^^,^^14^^,^^15^^,^^16^. MBT therapeutic techniques have been described^17^, such as rhythmic auditory stimulation, therapeutic instrumental music performance, melodic intonation therapy and musical mnemonic training. These techniques form the core of neurological music therapy^14^.

The effectiveness of interventions in the context of music-based interventions has been explained in terms of auditory-motor entrainment^18^ and sensorimotor coupling to temporally structured auditory input, along with recruitment of a striatal-thalamocortical system, involving the basal ganglia, thalamus, premotor, supplementary motor and dorsolateral prefrontal cortex^19^. This was found to have important effects with regard to connecting upper and lower body segments in coordinated movements, either symmetrically or asymmetrically (uni- or bi-manually)^20^.

Over recent years, MBT has been increasingly investigated in the context of neurological rehabilitation. MBT acts on motor skills and cognitive functions. A wide spectrum of MBT is known, such as writing music, singing songs from the light, classical and popular repertoires, rhythm-movement association (from physical relaxation to free gestures or structured rhythmic or dancing sequences), instrumental improvisation and listening to music tracks^17^. Some studies have highlighted the effectiveness of MBT for showing connectivity changes in different brain networks and enhancing motor recovery in terms of gait (velocity, cadence and stride length), upper-limb function and paresis, balance and mood functions^14^. Cognitive clinical effects have been observed in the domains of attention, memory, concentration and learning^21^^,^^22^, in which affective vocalizations have been shown to modulate attention via activation of prefrontal-limbic networks^23^. Studies have documented satisfactory results from programs using MBT among people diagnosed with stroke, Parkinson's disease, chronic aphasia, Alzheimer's disease, dementia and cerebral palsy^24^^,^^25^^,^^26^^,^^27^. On the other hand, the scientific evidence about MBT has only been systematically explored poorly among people with MS.

An understanding of the effects of MBT shown in relation to MS rehabilitation would clarify comprehension and could assist in clinical management and could serve as a basis for further research. The aim of the current study was to analyze the evidence for the effectiveness of MBT, within the therapeutic approaches to individuals diagnosed with MS.

## METHODS

A systematic review was conducted in order to provide an overview of the current state of evidence relating to MBT as an intervention for rehabilitation among people with MS. This study was conducted in accordance with the Preferred Reporting Items for Systematic Reviews and Meta-Analyses (PRISMA) guidelines^28^.

This study did not require animal or human participation; therefore, ethics approval was not required. The protocol for this systematic review was registered in PROSPERO, an international prospective register of systematic reviews (available at: http://www.crd.york.ac.uk/PROSPERO/), under the number: CDR42020143080 (University of York (UK), Centre for Reviews and Dissemination, National Institute for Health Research).

The systematic review was conducted by searching all papers registered in the following databases: BIOSIS, CINAHL, Cochrane, EBSCO, ERIC, Google Scholar, IBECS, LILACS, LISA (ProQuest), Medline, PEDro, PsycINFO (APA), Psychological & Behavioral, PubMed, SciELO, Scopus, SPORTDiscus and Web of Science. The subject descriptors proposed in the Medical Subject Headings (MeSH) and in the Health Sciences Descriptors (DeCS) were used: “Multiple sclerosis”, “music”, “music therapy”, “acoustic stimulation”, “acoustics”, “dance”, “dance therapy” and “rhythm” with crossing-referencing using the Boolean operators “AND” and “AND/OR”. No filter was used in the databases. Manual searches were also performed based on analysis of bibliographic references in previously selected articles. The search period ranged from the beginning of each database until July 2020.

Only studies that met the following criteria were included: (a) sample of individuals diagnosed with MS; (b) investigation of the effect of MBT as a treatment; (c) assessment of outcomes related to motor and/or non-motor factors; and (d) published study. The following characteristics of the studies were exclusion criteria: (a) presentation of samples with other neurological diagnoses associated with MS; (b) presentation of individuals with clinically isolated or radiologically isolated forms; (c) addressing a non-conservative intervention or pharmacological approach as a control; and (d) article classification as a review (systematic review or review of the literature), correspondence, editorial, conference abstract, observational study or book chapter. Only studies published in Portuguese and/or English were considered. There was no restriction on the year of publication of the study.

The procedures for selecting studies, extracting data and assessing methodological quality and risk of bias were independently developed by two reviewers. The results were compared and any disagreement was resolved through discussion. If there was no consensus between the reviewers, the opinion of a third reviewer would become necessary. For management of references, the Mendeley software was used, which enabling reference identification and control, especially in relation to the potential for duplication of references in different databases.

The selection and extraction of the data were done in line with the Cochrane recommendations^29^. The titles and abstracts of the studies were analyzed. Abstracts that met the criteria or those that needed further clarification were retained for complete review. Subsequently, the abstracts were analyzed in conjunction with the full texts of the articles. All outcomes were assessed at the conclusion of the MBT or in the light of its long-term effect.

The studies selected were analyzed in full text considering the following matters: 1. Referential characterization of the study; 2. Outline; 3. Sample; 4. Interventions; 5. Outcome measurements related to motor and non-motor factors; 6. Results; and 7. Conclusion. The effectiveness of MBT was analyzed in terms of the improvement of the outcomes that had been proposed in each study as a parameter.

The content of the studies selected was discussed based on the following topics: Investigation of the use of music as symptomatic and rehabilitation therapy for motor and non-motor disorders associated with MS; Analysis of the constituent characteristics and the mode of administration of the protocols for MBT, for rehabilitation of individuals with MS; Comparison between application of MBT and use of conservative noninvasive therapies, in care protocols for individuals with MS; Assessment of the methodological quality of the studies selected.

The methodological quality of each study was assessed using the PEDro scale (Physiotherapy Evidence Database), based on the Delphi list. This scale is composed of 11 items and scores 10. These items are scored as present (one point) or absent (zero point) and the total score is obtained by adding the item scores, thus, the maximum score of the PEDro scale is 10 points. Clinical trials with a PEDro score ≥6 points are classified as high quality; and <6 points, as low quality^30^. Studies with low methodological quality were not excluded, given that this was one of the aspects of these studies that was analyzed.

## RESULTS

### Studies included

**Figure 1:**
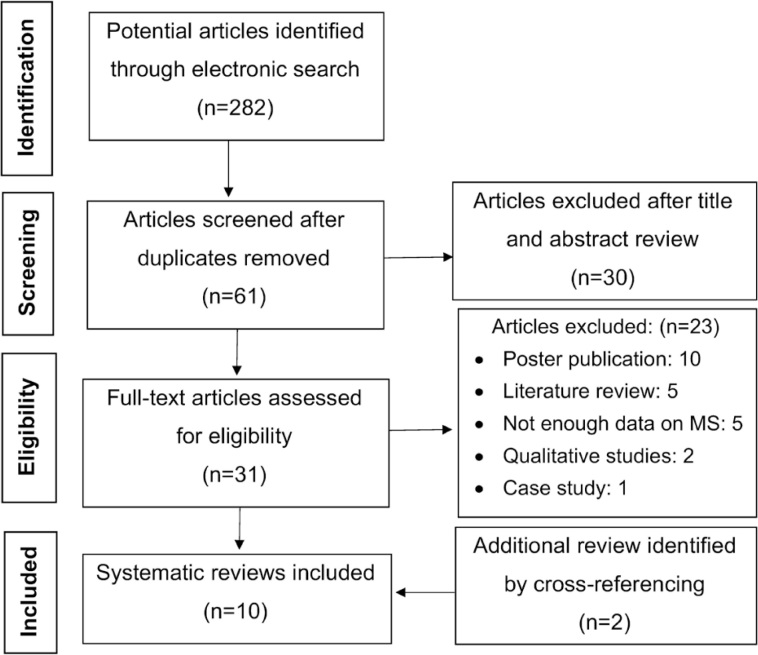
Flowchart of the systematic review analysis.

Using the search strategy, 282 citations published between the beginning of each database and July 2020 were identified, of which 10 were included in the systematic review (Figure 1). There was 100% agreement between reviewers regarding article eligibility. All the studies included are presented in Table 1. The outcomes assessed focused on both non-motor factors (anxiety, depression, quality of life, fatigue, mental fatigability, pain and cognition) and motor factors (balance, motor performance, walking endurance and dexterity). The studies used different assessment tools to evaluate these factors mentioned above.

**Table 1:** Characteristics of the studies included in the systematic review.

Author	Sample	Description	Outcomes
Aldridge et al.^37^	n=20 14 females: 6 males 29‒47 years old Clinical form: PP, SP EDSS: 2.5±1.5 Disease duration: 11 years	EG (n=10): Nordoff-Robbins approach: music-making on instruments or singing. (three blocks of music therapy in single sessions (8–10 per block) over the course of 1 year. CG (n=10): no intervention	Depression, anxiety and quality of life.
Conklyn et al.^31^	n=10 Gender: not mentioned 50.2±5.45 years old Clinical form: PP, SP, RR EDSS: not mentioned Disease duration: 16.6±10.43 years	EG (n=5): rhythmic auditory stimulation (mp3 playing). (20 min per day every day for 4 weeks) CG (n=5): no intervention	Gait performance and improvement of walking speed.
Gatti et al.^34^	n=19 12 females: 7 males 46±9.6 years old Clinical form: not mentioned EDSS: not mentioned Disease duration: not mentioned	EG (n=9): playing musical keyboard (turned on) (half an hour per day for 2 weeks) CG (n=10): playing music keyboard (turned off) (half an hour per day for 2 weeks)	Hand dexterity.
Geel et al.^1^	n=17 16 females: 1 male 29‒65 years old Clinical form: RR EDSS≤4.5 Disease duration: 3‒21 years	EG (n=7): dance (twice a week for ten weeks, 90 min per session) CG (n=10): art activities (poetry, paintings and photography) (twice a week for ten weeks, 90 min per session)	Fatigue and motor and mental fatigability, walking endurance and walking ability speed, balance, lower limb strength and manual dexterity.
Impellizzeri et al.^2^	n=30 11 females: 19 males 51.33±10.1 years old Clinical form: PP, SP, RR EDSS: 5±1.5 Disease duration: 9‒10 years	EG (n=15): NMT techniques, performed 3 times a week for 8 weeks. All the participants were subjected to the same amount of treatment (3 times a week for 8 weeks plus) CG (n=15): conventional cognitive rehabilitation (6 times a week for 8 weeks)	Cognitive abilities (attention, orientation, spatial abilities, memory and language), quality of life and depression.
Moore et al.^36^	n=38 30 females: 8 males 53.33±10.07 years old Clinical form: not mentioned EDSS: 4.88±1.26 Disease duration: not mentioned	EG (n=20): Music as mnemonic device using an adapted version of Rey's Auditory-Verbal Learning Test (AVLT) (immediate and after 15 min) CG (n=18): Hearing as mnemonic device using an adapted computer program using spoken words (immediate and after 20 min)	Learning and short memory.
Seebacher et al.^33^	n=101 85 females: 16 males 44.1±12 years old Clinical form: not mentioned EDSS: 2.0±1.5 Disease duration: not mentioned	EG (n=34): rhythmic cued motor imagery. (17 min of motor imagery, six times per week, for 4 weeks, with music) EG (n=34): metronome-cued motor imagery (17 min of motor imagery, six times per week, for 4 weeks, with metronome) CG (n=33): conventional physiotherapy (six times per week, for 4 weeks).	Walking speed, walking perception, fatigue and quality of life.
Seebacher et al.^32^	n=59 46 females: 13 males 44.4±15.4 years old Clinical form: not mentioned EDSS: 2.5±2.0 Disease duration: not mentioned	EG (n=19): music-cued and verbally cued motor imagery (six times per week, for 4 weeks) EG (n=20): music-cued MI (six times per week, for 4 weeks) CG (n =20): motor imagery (six times per week, for 4 weeks)	Fatigue and walking speed.
Thaut et al.^18^	n=54 38 females: 16 males 53.3±9.3 years old Clinical form: RR EDSS: 4.9±1.3 Disease duration: not mentioned	EG (n=27): music-assisted learning: single list of 15 words associated with melody of an originally composed song (sung). (single session of 10 trials, 20 minutes). CG (n=27): music-assisted learning: single list of 15 words associated with melody of an originally composed song (spoken). (single session of 10 trials, 20 minutes).	Verbal learning and short term memory improvements.
Young et al.^35^	n=81 42 females: 11 males 49.67±10.33 years old Clinical form: not mentioned EDSS: not mentioned Disease duration: not mentioned	EG (n=27): movement to music (multiple movement routines accompanied with music). (three 60-min per week for 12 weeks) EG (n=26): Yoga fit (three 60-min per week for 12 weeks) CG (n=28): no intervention	Mobility, balance, walking endurance, strength, fatigue and pain.

n: number of participants; PP: primarily progressive; SP: secondary progressive; RR: remitting-recurrent; EDSS: expanded disability status scale; EG: experimental group; CG: control group; NMT: neurological music therapy.

According to the PEDro scale, all the studies presented a high quality level (≥6). The majority of the studies met most of the criteria. None of the studies was blinded both to the subjects and to the therapists: this level of blinding would not have been possible due to the nature of the studies. Only three studies were blinded to the examiners (Table 2).

**Table 2:** Methodological quality score for each study (PEDro scale).

Reference	Item											Score
	Eligibility*	1-Randomized Allocation	2-Allocation Concealment	3-Similar prognosis	4-Blinding of participants	5-Blinding of therapists	6-Blinding of examiners	7-Outcome measurement	8-Intention to treat	9-Inter-group comparisons	10-Variability and precision	
Aldridge et al.^37^	0	1	1	1	0	0	0	1	1	1	1	7/10
Conklyn et al.^31^	1	1	1	1	0	0	0	1	0	1	1	6/10
Gatti et al.^34^	1	1	1	1	0	0	0	1	1	1	1	7/10
Geel et al.^1^	1	1	1	1	0	0	0	1	1	1	1	7/10
Impellizzeri et al.^2^	1	1	1	1	0	0	1	1	1	1	1	8/10
Moore et al.^36^	1	1	1	1	0	0	0	1	0	1	1	6/10
Seebacher et al.^33^	1	1	1	1	0	0	0	1	1	1	1	7/10
Seebacher et al.^32^	1	1	1	1	0	0	0	1	1	1	1	7/10
Thaut et al.^18^	1	1	0	1	0	0	1	1	0	1	1	6/10
Young et al.^35^	1	1	1	1	0	0	1	1	1	1	1	8/10

* Item not scored.

### Participants’ characteristics

The total number of participants studied was 429 (332 females and 97 males), among whom 253 were in the experimental group (EG) and 176 in the control group (CG). The sample sizes for the EG and CG in the different studies ranged from 5 to 34 individuals. All the participants were diagnosed with MS and were aged between 29 and 70 years old. The majority of the individuals sampled showed the clinical form of relapsing-remitting MS, with mean EDSS of 3.18±1.10 points and average MS duration of 11.82±1.10 years (Table 1). All the studies mentioned that participants were excluded when an exacerbation occurred three months or less before the onset of the study.

### Intervention characteristics

All the articles selected investigated the effect of MBT in relation to the standard physical treatment or no treatment. The EG underwent MBT sessions and the CG underwent conventional physiotherapy, MBT placebo or no intervention.

The duration of the sessions of EG and CG showed great variability between studies. Most sessions lasted between 15 and 90 minutes, often between 2 and 3 times a week, in protocols lasting for between 2 and 60 weeks.

The treatment protocols based on interventions involving music used strategies that included application of activities relating to dance, rhythm, singing, memorization of parts of music through spoken or sung speech, mental imaging associated with music or playing musical instruments.

The group of participants who were allocated to the CG underwent several strategies such as maintaining their daily activities (without the intervention itself), developing artistic activities (poetry or painting) that did not involve music and/or musical elements, cognitive rehabilitation, yoga exercises, conventional physiotherapy, motor imagery or playing musical instruments without sound. All of the studies reported that there were no adverse events.

### Music-based therapy interventions and outcomes

#### Rhythmic auditory strategies: gait, fatigue and quality of life

Conklyn et al.^31^ tested a four-week home-based walking program with rhythmic auditory stimulation, among 10 MS patients. They found that there was a significant improvement in gait parameters (doubled support time and walking speed) after two weeks of treatments. In a home-based pilot study, Seebacher et al.^32^ applying music-cued and verbally cued motor imagery in one group and music-cued motor imagery in another, and comparing both of these with a CG in which only motor imagery was applied. They found improvements relating to fatigue follow-up, compared with baseline, in both treatment groups. In another study, the same authors^33^ applied rhythmic-cued motor imagery techniques to a large sample of MS patients. They confirmed the improvements in walking, fatigue perception and quality of life after a treatment applied six times per week, for 4 weeks (Cohen's d=0.6).

#### Playing musical instruments: dexterity

Gatti et al.^34^ evaluated the efficacy of musical keyboard playing on hand function improvement in a group of 19 MS patients, with training for half an hour per day for 2 weeks. Half of the patients played a turned-on musical keyboard using finger movements (audio on); the other half used a turned-off keyboard (audio off). The whole program consisted of 46 exercises applied according to increasing levels of difficulty, which were firstly demonstrated by a music therapist three times. The results were valuable for improvement in hand dexterity (assessed by means of the nine-hole peg test) and in perceived hand functional use, with a difference between the two groups (Cohen's d=1.66).

#### Dance strategy: balance, coordination, fatigue, mental fatigability, walking, strength, cognitive, quality of life and pain

Only two studies^1^^,^^35^ reported on the effect of dance. Geel et al.^1^ assessed this effect in relation to fatigue and mental fatigability, balance, walking ability, lower limb strength and manual dexterity. Comparing EG (dance) and CG (art activities), Geel et al.^1^ showed that there were significant improvements in the level of fatigue, cognitive capacity and coordination for the EG. There was no significant change in mental fatigability and health-related quality of life.

Young et al.^35^ assessed the effects of MBT in comparison with adapted yoga. The primary outcomes were improvement in mobility and balance, walking endurance and lower extremity functional strength. The secondary outcomes were reduction of fatigue and pain. Young et al.^35^ showed that significant improvements with moderate effect sizes occurred in relation to balance (Cohen's d=0.7) and walking time performance (Cohen's d=0.6), with a trend towards fatigue reduction, in a group of participants who underwent MBT, compared with a CG.

#### Music mnemonics: cognition, mood and behavioral and verbal communication

It has been highlighted that musical mnemonic devices facilitate verbal learning and short-term memory among MS patients. Moore et al.^36^ used music as a mnemonic device to test learning and memory ability among 38 MS patients. They were divided in two groups: patients who learned words through music versus patients who learned words through speech. Even though the results did not show any significant differences in memory between the groups, there was a significant correlation between auditory verbal learning test results and clinical measurements in the music group, in comparison with speech group.

Thaut et al.^18^ investigated the neural correlates of brain plasticity during verbal memory training, with use of music mnemonics. Their results showed that speech and order memory were better under musical conditions than under spoken conditions, with stronger bilateral frontal alpha learning-related synchronization in the first group.

Using the Nordoff-Robbins approach (active role of both patients and musical therapist regarding vocal improvisation or playing instruments, without any previous musical education), Aldridge et al.^37^ performed three blocks of MBT in a single session over a one-year period, in a group of 20 MS patients. Despite no significant differences, the authors found improvements of medium effect size on the scales measuring self-esteem (Cohen's d=0.5), depression (d=0.63) and anxiety (d=0.63), which thus suggested that MBT can influence the behavior and mood aspects of MS patients.

#### Neurological music therapy: cognitive function, emotional status, motivation, mood and quality of life

Neurological music therapy (NMT) is a new integrative therapeutic approach that was presented in only one article^2^ in this review. A typical NMT technique is based on an associative network theory of mood and memory. This suggests that when an event or some information is processed, neural connections are established together with other elements (emotional status, odors and environmental background) of that event, and are stored as nodes in memory. Later, this neural node can be activated that musical stimuli^38^.

Impellizzeri et al.^2^ conducted a study with a sample composed of 30 MS participants. These were randomly in 2 groups: the CG underwent conventional cognitive rehabilitation (CCR) (6 times a week for 8 weeks); and the EG underwent CCR plus NMT techniques (3 times a week for 8 weeks). In particular, the EG got better results for cognitive function, with regard to selective reminding test long-term storage (p<0.000), long-term retrieval (p=0.007) and delayed recall of the 10/36 spatial recall test (p=0.001), in comparison with the CG. Moreover, the improvement in emotional status, motivation, mood and quality of life (with regard to the mental component; p<0.000 was more evident in the EG.

## DISCUSSION

There has been an exponential increase in knowledge about MBT as a therapeutic complementary approach for MS. Its efficacy is continually increasing, which makes MBT more reliable and precise. The analysis on this systematic review revealed that MBT provides strong clinical potential for rehabilitation of non-motor and motor factors among people with MS.

This review included studies published between 2005 and 2020. However, there is a lack of studies about MBT and its efficacy with good methodological standards and with analysis on specific outcomes relating to MS rehabilitation. Additionally, it can be hypothesized that the effect relating to degenerative diseases like MS is underestimated, given their progressive nature. However, since the intervention period reported was relatively short and these studies included a CG, it is very unlikely that disease progression could have greatly hampered correct interpretation of the results^14^.

Although music therapy has been recognized as a profession in the healthcare field in the United States since 1956^39^, there is an urgent need in Brazil for music therapy to be recognized and strengthened as a healthcare profession, thereby becoming clearly differentiated from musical activities that relate to relaxation and leisure. Only limited scientific evidence supports the use of this clinical intervention among MS patients^40^. The rehabilitative effect of music in relation to neurological disorders is linked to changes in brain neuroplasticity and neural activation^3^^,^^24^^,^^41^, but the specific mechanism still remains unknown.

A variety of ways to use MBT in strategies for rehabilitation of people with MS and to measure the outcomes was observed. The studies reviewed here evaluated MBT as a tool for rehabilitative purposes, through active techniques (musical improvisation through playing an instrument or singing) and passive techniques (music imagery and listening to music). In both types of technique, the aim was to build up a musical relationship between patient and therapist, so as to promote motor and cognitive recovery. This was shown by Vinciguerra et al.^42^ through their exploration of the role of music therapy in MS.

In this current review, to facilitate interpretation of the effects from interventions, and for simplicity of explanation, the findings from the studies selected were grouped into four clusters, according to the content of the music-based strategy used: (1) Rhythmic auditory; (2) Playing musical instruments; (3) Dance strategy; and (4) Neurological music therapy. The studies focused more on analyzing the evidence for the effect of music-based therapy on motor outcomes.

All the studies yielded promising positive results in relation to both the motor and the non-motor outcomes that were assessed. The music interventions were mostly superior to conventional therapy or no intervention. As such, this review provides support for implementing elements of music-supported rehabilitation in the field of neurology. However, it should be noted that the outcome measurements selected were specific to the outcomes that was targeted for assessment. Therefore, the results are not generalizable for all symptoms that give rise to impairment in the MS population.

Regarding the rhythmic auditory strategy, improvement of gait and reduced fatigue levels were shown. Impaired walking speed due to gait limitations or fatigue plays a crucial role in reducing the level of ability to perform activities of daily living among people with MS^42^. Rhythmic auditory stimulation provides improvement of the intrinsic rhythmic movements of gait and the coordination processes. This may be related to involvement of different motor brain areas, such as the cortex, cerebellum and spinal tract^31^^,^^43^. Vinciguerra et al.^42^ discussed the way in which music also exploits the relationship between body language and sound, such as interaction between perception and action. In this context, rhythmic auditory stimulation, more than simple metronome cues, has been developed to promote walking speed and distance among people with motor limitations.

Analysis on the intervention “playing musical instruments” showed that this strategy focused on mobility outcomes, given that it directly involved an activity. This is an active musical experience that, through including repetitive movements, involves different cerebral regions across a multisensory stimulation effect (visual, vestibular, tactile and proprioceptive), thus promoting functional recovery of the hands^44^.

Cognitive impairment affects 70% of people with MS^45^. Some deficits, especially relating to memory, attention and information processing speed, are present from the early stage of the disease. So far, there is a lack of effective drugs or other treatments that might help to cope with these cognitive dysfunctions. There is a lack of strategies for improving cognition in MS and the ways in which this matter has been approached have shown conflicting results. In this review, one randomized clinical trial that applied the NMT intervention was seen: this is a new approach that can be included among MBT strategies.

Research promoting music as a mnemonic tool for improving neuronal plasticity of the temporal regions and oscillatory network synchronization in the prefrontal area resulted in better learning and memory performance^18^^,^^42^. However, Moore et al.^36^ reported that the rhythmic and melodic phrases within music did not seem to directly support music as a mnemonic device in facilitating learning processes and information retrieval. This may have been because recognition tasks are not the most appropriate method for measuring the effectiveness of temporal structures in retrieving learned information. Those authors explained their results through the assumption that the participants did not have enough time to memorize the information and that the music could also have represented a distractor element. Nonetheless, it was also true that in the same study, the analysis on correlations in the music group showed that less cognitively impaired MS patients gained more benefits from music mnemonics, in relation to learning and memory processes. Music-based interventions can be used as dual-task training, apart from providing simple training for motricity or cognition^14^.

To demonstrate the components of the dual task, using MBT, the action of physically producing music is performed simultaneously with auditory stimulus processing or visual stimulus processing. This is relevant, given that large cognitive-motor interferences resulting from dual tasks have been shown in many neurological conditions and have been correlated with the risk of falls^14^.

MBT was also associated with approaches for improving emotional aspects and quality of life of MS patients. The physiological explanation for this phenomenon relates to the effect on neurohormonal circuits such as activation of the mesolimbic dopaminergic system (nucleus accumbens, hippocampus and amygdala)^23^^,^^46^. Music is able to activate the parasympathetic nervous system, in contrast to the sympathetic system, thereby promoting cytokine secretion and changes in catecholamine levels^47^. Music represents part of our genetic heritage, a kind of universal language focused on the relationship between the language of the body and that of sound, which is able to reduce stress and preserve mental health conditions. These data are promising and lead to consideration of the use of MBT as a drug-free treatment for supporting individuals’ coping strategies in MS^42^.

There were important limitations to this study. The studies could have provided more details and could have demonstrated all the results in the publications than they did. For example, some studies did not show basic data like the EDSS score or the clinical form of MS. For this reason, it was not possible to analyze the conditions of the sample at the baseline and hence the real dimension of the MBT effects on this specific population. There is a lack of meta-analysis in relation to MBT, in comparison with other rehabilitation approaches. This is because of the heterogeneity of samples, follow-up periods, modalities of music intervention, modes of administration of MBT interventions, more than one experimental group and numbers of participants included in each study.

Indeed, MBT effects can be underestimated, given the progressive course of MS disease, and the role of this intervention still appears unclear. Blinding of participants or therapists was not feasible, but may still represent a risk of bias. Although we searched extensively, we may have missed relevant studies. Because MBT is a simple and low-cost intervention, it deserves to be studied in larger samples of MS patients, with a wide spectrum of clinical disability and appropriate approaches that are chosen such that they are tailored to individual clinical outcomes.

Although the clinical effects of music-based interventions are promising, as shown in this review, further research is needed in order to better understand the principles of how music interacts with motricity or cognition. To further investigate this hypothesis, it is important to apply comparative experimental designs and to use neuroimaging techniques to investigate the neurophysiological processes of music-based interventions. Further research is needed in order to combine effective rehabilitation approaches with appropriate study designs, outcome measurements, types and intensities of modalities and cost effectiveness of these interventions.

MBT is a safe and effective approach for clinical rehabilitation of MS patients that leads to positive results regarding motor function. However, regarding mental fatigability and memory, the data were conflicting and the evidence was unclear. Although generalization of these findings may be restricted by the small sample size, this systematic review showed that MBT can be indicated for improving motor factors, even in a neurodegenerative disease like MS. It is essential to better define these approaches using elements of music that were cited as strategies in this current review. It is relevant to identify more standardized methods to apply in each clinical context. The role of music needs to be better understood and included in a multidisciplinary approach for different MBT settings. This can help to further explain the role of music in relation to brain neuroplasticity changes and thus confirm the strong relevance of MBT in clinical practice.
